# Biologically inspired approaches to enhance human organoid complexity

**DOI:** 10.1242/dev.166173

**Published:** 2019-04-16

**Authors:** Emily M. Holloway, Meghan M. Capeling, Jason R. Spence

**Affiliations:** 1 Department of Cell and Developmental Biology, University of Michigan Medical School, Ann Arbor, MI 48109, USA; 2 Department of Biomedical Engineering, University of Michigan College of Engineering, Ann Arbor, MI 48109, USA; 3 Department of Internal Medicine, Division of Gastroenterology, University of Michigan Medical School, Ann Arbor, MI 48109, USA; 4 Center for Organogenesis, University of Michigan Medical School, Ann Arbor, MI 48109, USA

**Keywords:** Human development, Organoids, Stem cells

## Abstract

Organoids are complex three-dimensional *in vitro* organ-like model systems. Human organoids, which are derived from human pluripotent stem cells or primary human donor tissue, have been used to address fundamental questions about human development, stem cell biology and organ regeneration. Focus has now shifted towards implementation of organoids for biological discovery and advancing existing systems to more faithfully recapitulate the native organ. This work has highlighted significant unknowns in human biology and has invigorated new exploration into the cellular makeup of human organs during development and in the adult – work that is crucial for providing appropriate benchmarks for organoid systems. In this Review, we discuss efforts to characterize human organ cellular complexity and attempts to make organoid models more realistic through co-culture, transplantation and bioengineering approaches.

## Abstract (precis)

**Summary:** This Review discusses the advances in human organoid technologies to improve organoid complexity, through co-culture, transplantation and bioengineering approaches.

## Introduction

The intricacies of embryonic development have captivated developmental biologists for centuries. The process by which a single cell undergoes gastrulation, morphogenesis and organogenesis to ultimately give rise to a fully formed body still captures the curiosity of scientists. This curiosity has been partially satisfied by our ability to interrogate developing animal model systems such as worms, flies, frogs, fish, chicks and mice. These systems have been used because they are accessible to the human eye and can be experimentally manipulated. Still, despite having access to developmental model systems, we refer to them as ‘models’ because scientists ultimately look to these systems to gain insights into human biology. In the recent past, however, new experimental systems have allowed us to further our understanding of human development, including the ability to culture human embryonic stem cells (Thomson, 1998) or to reprogram human somatic cells to an embryonic state, termed induced pluripotency (Takahashi et al., 2007). These systems serve as platforms to study the earliest stages of human development (Deglincerti et al., 2016; Shahbazi et al., 2017; Shao et al., 2017; Takashima et al., 2014).

Given the high degree of evolutionary conservation and the exceptional robustness of embryonic development, concepts and insights that have been gained from model systems have been used in attempts to model ‘human development in a dish’ using human embryonic and induced pluripotent stem cells (iPSCs), together known as hPSCs, *in vitro*. Human stem cells have been differentiated into complex three-dimensional (3D) structures, referred to as ‘organoids’ because of their organ-like properties, which have been ideal human platforms to study development, regeneration and disease. hPSC-derived organoids circumvent the scarce availability and accessibility to primary human tissue samples and possess cell types that are found in the native *in vivo* organ environment. 3D *in vitro* human platforms have been developed from hPSCs for a variety of organ systems including (but not limited to) lung (Chen et al., 2017; Dye et al., 2015; Konishi et al., 2016; McCauley et al., 2017; Miller et al., 2019; Miller et al., 2018), stomach (McCracken et al., 2014), esophagus (Trisno et al., 2018; Zhang et al., 2018b), intestine (Múnera et al., 2017; Spence et al., 2010), kidney (Taguchi et al., 2014; Takasato et al., 2015), liver (Takebe et al., 2013), eye (Eiraku et al., 2011; Nakano et al., 2012), heart (Hoang et al., 2018), inner ear (Koehler et al., 2017) and brain (Jo et al., 2016; Lancaster et al., 2013; Muguruma et al., 2015; Qian et al., 2016; Sloan et al., 2018). Importantly, organoids can also be derived from tissue-resident stem cells that are isolated from primary human tissue donors for several organ systems including (but not limited to) intestine (Sato et al., 2011), stomach (Bartfeld et al., 2015), liver (Hu et al., 2018; Huch et al., 2015) and lung (Miller et al., 2018; Nikolić et al., 2017; Rock et al., 2009; Sachs et al., 2019).

Organoids have already proven to be an excellent platform for drug screening (Czerniecki et al., 2018; Dekkers et al., 2013; Watanabe et al., 2017) and may hold promise for organ replacement therapies; however, their major strength currently comes as a discovery tool to understand human development and disease. Despite the remarkable progress that has been made towards understanding human development, organoid models are, in many respects, still fairly primitive, lacking important features that are found in the native tissue. Thus, studies have now shifted toward improving organoid models, increasing complexity and making them more organ-like, in order to understand how complex tissues form. Co-culture systems and transplantation into immunocompromised murine hosts are two methods that are currently being used to increase complexity in organoid models and to promote organoid maturation into an adult-like tissue. Still, organoid cultures are heterogeneous and variable. Therefore, in addition to increasing complexity of organoids, recent work has focused on using engineering strategies such as chemically defined 3D hydrogels to improve experimental control and reduce variability within organoid cultures.

As organoid cultures are quickly advancing in complexity, ensuring that these models faithfully recapitulate the *in vivo* tissue counterpart is crucial. Most of the initial characterization of organoid cultures has depended on comparative analysis with animal models. Recent work has uncovered important species-specific differences that could limit this approach (La Manno et al., 2016; Miller et al., 2018; Nikolić et al., 2017). With this in mind, ongoing efforts to benchmark all cells in the human body and across the human developmental continuum will play a crucial role in providing needed references with which to compare *de novo* formed organoids (Gerrard et al., 2016; Regev et al., 2017; Rozenblatt-Rosen et al., 2017). Benchmarking efforts are already aiding in more accurate characterization of organoids (Camp and Treutlein, 2017; Camp et al., 2015; Kinchen et al., 2018; La Manno et al., 2016; Menon et al., 2018; Pollen et al., 2015; Quadrato et al., 2017; Zhong et al., 2018). Moreover, organoid-to-tissue comparison at single cell resolution allows for unprecedented insights into cellular heterogeneity and lineage relationships of both primary tissue and organoid cultures (Camp et al., 2015, 2017; Combes et al., 2019). In the case of cerebral organoids, it was found that the majority of genes are expressed at similar levels in cerebral organoids as in the human fetal cerebral cortex (Camp et al., 2015). Benchmarking liver iPSC-derived hepatocyte-like liver organoids against both human fetal and adult tissues demonstrated that hepatocyte populations derived *in vitro* were more closely aligned with fetal tissue (Camp et al., 2017). Integrating information from unbiased profiling of primary human tissue specimens and organoid cultures will allow for thorough assessments of organoid culture advancements towards generating a complete tissue in a dish.

In this Review, we discuss recent efforts to improve organoid complexity to more closely resemble native tissue, while at the same time improving robustness, reproducibility and control over the experimental system. We also discuss unique applications of organoid technologies to uncover novel biological insights into human development and disease, and highlight challenges that organoid systems could address in order to realize their full potential.

## Improving complexity of organoid models through co-culture

The process of directing differentiation of pluripotent stem cells is achieved by mimicking developmental cues in a temporally controlled fashion through the addition of various recombinant proteins and small molecules to control lineage specification. Resulting organoids can possess diverse cell types from multiple germ layers (Chen et al., 2017; Dye et al., 2015; McCracken et al., 2014; Múnera et al., 2017; Spence et al., 2010; Taguchi et al., 2014; Takasato et al., 2015; Zhang et al., 2018b). However, crucial lineages are often absent in organoids. For example, in organoid models of the intestine, vascular, neuronal and immune lineages appear to be absent (Finkbeiner et al., 2015b; Spence et al., 2010; Watson et al., 2014; Wells and Spence, 2014). In addition, regardless of culture duration, pluripotent stem cell-derived organoids fail to mature *in vitro*, and remain similar to fetal tissue (Aurora and Spence, 2016; Camp et al., 2015; Chen et al., 2017; Finkbeiner et al., 2015b; Hrvatin et al., 2014; Takasato et al., 2015). Recent efforts have focused on increasing the complexity of organoid models through co-culture with missing cellular components as well as with microbes. Below, we discuss examples of how co-cultures have been used to introduce cellular components or microorganisms into organoid platforms to improve the complexity of organoid models and to study host-microbe interactions.

### Introducing enteric nerves into human intestinal organoid models

Intestinal organoids exemplify a system that has benefited from co-culture with both missing human cell types as well as with microbes. Human intestinal organoids (HIOs) are generated from hPSCs via directed differentiation that results in 3D structures consisting of intestinal epithelium surrounded by mesenchymal cells (Spence et al., 2010). After several weeks of culture in a 3D extracellular matrix (ECM) or in bioengineered hydrogels (Capeling et al., 2019; Cruz-Acuña et al., 2017), HIOs possessed multiple epithelial and mesenchymal lineages that are found in the developing human intestine (Aurora and Spence, 2016; Finkbeiner et al., 2015b; Spence et al., 2010; Watson et al., 2014; Wells and Spence, 2014). Although hPSC-derived HIOs possessed many of the key cell types that are found in the native intestine, they lacked an enteric nervous system (ENS), immune cell lineages and vasculature (Finkbeiner et al., 2015b; Wells and Spence, 2014).

The ENS, which develops from ectoderm-derived vagal and sacral neural crest cells (Burns, 2005; Sasselli et al., 2012), is responsible for a diverse set of functions that include gastrointestinal motility as well as regulation of blood flow, regulation of the epithelial barrier and fluid movement (Furness, 2012). The importance of the ENS is underscored by serious disorders that are caused by an absent or damaged ENS, including Hirschsprung's disease and Chagas disease, both of which demand intense medical and surgical intervention (Furness, 2012).

**Fig. 1.**
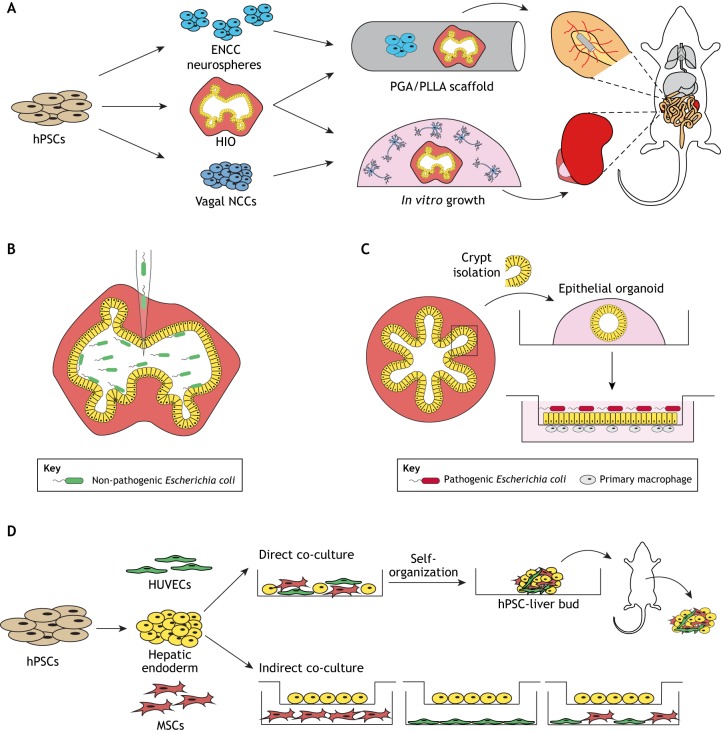
**Increasing organoid complexity through co-culture.** (A) Approaches to incorporate enteric nerves into HIOs through co-culture. Top: HIOs were seeded with hPSC-derived ENCC spheres onto PGA/PLLA scaffolds and immediately transplanted into the intestinal mesentery. Bottom: HIOs were co-cultured with hPSC-derived vagal NCCs *in vitro* and were transplanted beneath the kidney capsule of an immunocompromised mouse for further maturation. (B) Microinjection is a common approach to introduce various microbes into the lumen of 3D gastrointestinal organoids. For example, a non-pathogenic strain of *E.* *coli* is depicted being injected into the lumen of hPSC-derived HIOs. (C) 2D monolayer cultures have been established from 3D biopsy-derived human intestinal and colon epithelial organoids. These 2D systems allow for easy access to both apical and basal regions of organoid epithelium. Recently, microbial pathogen-immune crosstalk was assessed across this polarized primary organoid-derived epithelium. (D) Liver bud formation can be achieved through mesenchyme-endothelial-hPSC-derived hepatic endoderm co-culture, which undergoes 3D self-organization after only a few days in culture. Transplantation and transwell culture systems have been used to further study hepatocyte maturation within the liver buds.

Engineering an HIO with an ENS would increase HIO functionality, enable disease modeling and allow researchers to study the mechanisms that guide human ENS development. Vagal-like neural crest cells (NCCs) are the predominant precursors for ENS lineages of the small intestine (Sasselli et al., 2012). Vagal-like NCCs were generated from hPSCs through directed differentiation and incorporated into HIOs through direct co-culture (Workman et al., 2016) (Fig. 1A). NCCs co-cultured with HIOs demonstrated multilineage differentiation potential into neurons and glia, lineages that are not apparent when HIOs are cultured independently. Protein marker analysis demonstrated the presence of diverse neuronal fates including dopaminergic neurons, sensory neurons, interneurons and excitatory neurons. In addition, these neurons were capable of responding to calcium stimulation, demonstrating neuronal function. However, ENS organization resembled an immature, fetal-like ENS. Global transcriptional profiling of co-cultured HIOs and HIOs alone revealed effects of ENS incorporation on HIO epithelial development. There was a notable increase in expression of genes that are characteristic of intestinal stem cells and transit amplifying cells, at the expense of differentiated absorptive and secretory intestinal cell types, suggesting that exposure to ENS components influenced the differentiation trajectory of HIOs. Notably, ganglia organization and NCC differentiation into the inhibitory neuronal fate were induced upon *in vivo* transplantation and growth beneath the kidney capsule in an immunocompromised mouse. Importantly, transplanted HIOs with an ENS had the ability to undergo peristaltic-like contractions, indicating that they had gained gastrointestinal motility. One caveat to this model was that the neurons failed to associate with the HIO epithelium, whereas in the native intestine the ENS forms connections with the enteroendocrine cells of the epithelium to convey nutrient sensing information (Bohórquez et al., 2015).

Recently, this neuro-epithelial interaction in HIO-ENS co-cultures was accomplished through the use of enteric neural crest cell (ENCC) spheres (Fattahi et al., 2016), which are 3D progenitor cultures that are capable of differentiating into the ENS lineages, along with hPSC-derived tissue-engineered small intestine (Finkbeiner et al., 2015a; Schlieve et al., 2017) (Fig. 1A). The HIO-ENS co-culture platform has also been used to interrogate the molecular mechanisms of Hirschsprung's disease, a developmental disease that is characterized by aganglionosis in the distal intestine thought to arise from improper migration of NCCs (Furness, 2012; Workman et al., 2016). Collectively, these works improved organoid complexity by introducing an ENS and developing platforms to begin studying human intestinal motility and associated human ENS neuropathies.

### Using organoids to model host-microbe and immune interactions

Human organoid models that are derived from both primary tissue and hPSCs have made it possible to study host-microbe interactions that were challenging because of species-specificity that led to poor infection and replication rates in animal models (Lazear et al., 2016). For example, co-cultures of cerebral organoids and neurospheres (3D aggregates of hPSC- or primary-derived neuronal stem and progenitor cells) with Zika virus have provided important mechanistic insights into the pathology of this harmful developmental infection in a human model system (Gabriel et al., 2017; Garcez et al., 2016; Muffat et al., 2018; Qian et al., 2016; Watanabe et al., 2017). Zika virus severely affects brain development in the first and second trimester following viral exposure, and is correlated with severe developmental defects and microcephaly (Faria et al., 2016). Moreover, this virus does not naturally infect rodents, further highlighting brain organoids as an ideal model system to understand viral pathogenesis (Lazear et al., 2016). Consistent with clinical observations, initial experiments infected cerebral organoids with Zika virus and found that Zika preferentially targeted neuronal progenitor populations (Garcez et al., 2016; Qian et al., 2016). Zika infection induced increased levels of apoptosis while suppressing proliferation, which resulted in smaller organoids that displayed many attributes of microcephaly. Cytotoxicity is predominantly restricted to neuronal progenitors, but the virus has been shown to infect a broader variety of hPSC-derived neuronal lineages including microglial precursors, hinting at a potential infection mechanism (Muffat et al., 2018). However, despite their utility for studying viral pathogenesis, cerebral organoids do not fully recapitulate the developing human brain as they lack crucial components, including immune cells and a vascular system. However, microglia-like cells, the resident innate immune cells of the central nervous system, have recently been derived from hPSCs and co-cultured with human cerebral organoids in an effort to introduce further complexity to the current brain organoids (Abud et al., 2017; Muffat et al., 2016, 2018; Pandya et al., 2017). These studies demonstrate the utility of human cerebral organoid model systems to study mechanisms of infection, tissue response and neurological disease.

Another context in which organoids are being used to model complex interactions is in the gastrointestinal system. The human gastrointestinal epithelium serves a complex role, including nutrient uptake, forming a tight physical barrier to protect against environmental antigens (i.e. food products) and commensal or pathogenic microorganisms within the gut (Peterson and Artis, 2014). The epithelium must also effectively communicate with the immune system to maintain barrier homeostasis, and dysregulation of this fine balance can lead to diseases such as inflammatory bowel disease (Henderson et al., 2011; Martini et al., 2017; Okumura and Takeda, 2017). There have, therefore, been efforts towards co-culturing gastrointestinal tissue with microbes to recapitulate host-microbe interactions in the gut and to model epithelium-immune interactions. Both hPSC-derived gastrointestinal (stomach, intestinal) organoids (Finkbeiner et al., 2012; Hill et al., 2017a; Leslie et al., 2014; McCracken et al., 2014) and primary tissue (i.e. biopsy-derived) epithelial organoids (Bartfeld et al., 2015; Bertaux-Skeirik et al., 2015; Heo et al., 2018; In et al., 2016; Saxena et al., 2017) have been used to model these complex host-microbe-immune interactions (Fig. 1B,C; Box 1).

Box 1. Modeling pathogenic infections using organoids Organoids spanning a variety of systems have been used to interrogate epithelial responses to microbial infections. For example, primary tissue-derived gastric organoids have been used to study *Helicobacter pylori* infection (Bartfeld et al., 2015; Bertaux-Skeirik et al., 2015). To better understand host response to initial infection, gastric organoids were microinjected with *H. pylori* and microarray analysis was performed to identify differentially expressed genes after only 2 h of infection. This approach identified the upregulation of several NF-kB target genes upon *H. pylori* infection (Bartfeld et al., 2015). Recently, microinjection approaches have also been used to model *Cryptosporidium* infection in primary tissue-derived human intestinal and lung organoids (Heo et al., 2018). Epithelial response has also been studied using 2D organoid-derived monolayer culture conditions. These 2D systems have been used to study *H. pylori* infection of gastric epithelium as well as norovirus and pathogenic *E. coli* infection of intestinal epithelium (Boccellato et al., 2018; Ettayebi et al., 2016; In et al., 2016; Rajan et al., 2018).

HIOs have also been used to model microbial colonization of the immature gut that takes place at birth (Bäckhed et al., 2015; Hill et al., 2017a,b; Koenig et al., 2011; Palmer et al., 2007; Stewart et al., 2018; Vatanen et al., 2018). The 3D growth of organoids makes it technically challenging to access the apical surface of the epithelium, which faces the inside of the enclosed lumen and requires cumbersome and labor-intensive microinjection. Although laborious, microinjection was shown to be an effective strategy to access the enclosed lumen of HIOs (Hill et al., 2017a; Hill et al., 2017b; Leslie et al., 2014). In the case of commensal *Escherichia coli*, HIOs supported colonization for over a week, which allowed for careful examination of epithelial changes throughout microbial colonization (Hill et al., 2017a). Transcriptional profiling demonstrated upregulation of key genes that are involved in the epithelial barrier, innate immunity, hypoxia and tissue maturation over the course of a 4-day exposure to *E. coli*. Interestingly, peak expression of these sets of genes was temporally regulated – expression of gene classes that are involved in the epithelial barrier peaked early after infection and genes that are associated with tissue differentiation and maturation peaked several days later. This was the first work of its kind that used hPSC-derived intestinal organoids to study commensal microbial colonization of the human small intestine. Future studies can employ similar technical approaches using different microbial strains to broaden our understanding of microbiome interactions during early human development.

Notably, efforts have defined conditions in which 3D epithelial-only gastric and intestinal organoids can be plated as two-dimensional (2D) monolayers while retaining the cellular heterogeneity and apical-basal polarity that is present in standard 3D environments (Boccellato et al., 2018; Foulke-Abel et al., 2014; In et al., 2016; Kozuka et al., 2017; Thorne et al., 2018; Wang et al., 2015). Notably, both 2D and 3D models of the gastrointestinal system have been used to study epithelial responses to a spectrum of pathogenic microbes (Box 1). In the future, these systems may also be suitable for modeling diseases such as necrotizing enterocolitis, a devastating disease that is caused by excessive inflammation in premature infants (Senger et al., 2018). Recent work leveraged an epithelial organoid-derived monolayer culture system that was established from human intestinal biopsies to study crosstalk between macrophages, a prevalent resident immune population in the intestine, and the intestinal epithelium (Noel et al., 2017). When human macrophages were seeded along the basal surface of these monolayer cultures, barrier properties, including epithelial cell height and transepithelial electrical resistance, were enhanced, indicating a strengthened barrier upon co-culture with macrophages. Moreover, when pathogenic strains of *E. coli* were introduced to the apical surface of the co-cultures, macrophages were able to sense the pathogen and evoke phagocytic defense machinery (Fig. 1C). In addition, macrophages underwent morphological changes in response to the pathogenic *E. coli*, extending projections across the epithelial barrier in order to survey the luminal environment. Collectively, these data provide new insights into macrophage-epithelial crosstalk in the human intestine. This approach is an improvement from the previous use of immortalized intestinal cell lines, as these organoids contain all of the epithelial cell types found within the native intestine. This system can be applied in future work to incorporate new and/or additional cell types to improve our understanding of immune-epithelial crosstalk during intestinal development. In addition, primary tissue-derived organoids are being used to study tumor microenvironments and cancer immunotherapies (Dijkstra et al., 2018; Neal et al., 2018). These works demonstrate the progress in the introduction of new cell lineages and allow for precise study of crosstalk in a scalable, modular *in vitro* environment.

### Complex liver bud formation through mesenchyme-endothelial-hepatocyte co-culture

Co-culture systems have also enhanced models of the developing liver. Previous studies have derived liver hepatocyte-like cells in 2D from hPSCs; however, the resulting cells were immature and did not possess the full complement of mature hepatocyte function (Agarwal et al., 2008; Cai et al., 2007; Duan et al., 2007; Song et al., 2009; Sullivan et al., 2009; Si-Tayeb et al., 2010; Touboul et al., 2009). Moreover, hPSC-derived hepatocyte-like cells engrafted into immunocompromised mice with low efficiency (Azuma et al., 2007; Carpentier et al., 2014; Hannoun et al., 2016; Haridass et al., 2009; Liu et al., 2011). Considering the immature phenotypes of 2D hPSC-derived hepatocytes and the difficulty in engrafting, there have been recent efforts to generate more complex liver organoid models with the aim of improving hPSC-derived hepatocyte function by recreating a more complex environment through co-culture. In mice, endothelial cells have been identified as an imperative cell type that drives liver organogenesis (Matsumoto et al., 2001). It was, therefore, hypothesized that co-culture with endothelial cells and mesenchymal precursors with hepatic endoderm would more accurately represent *in vivo* development, and result in improved hepatocyte differentiation (Takebe et al., 2013). By independently culturing these three cell types and then combining them together in 2D culture, the co-cultures condensed to form an immature 3D liver bud *in vitro* after only a few days in the dish. These approaches have led to the development of complex ‘human liver bud organoids’, made of hPSC-derived hepatic endoderm (hPSC-HE), human umbilical vein endothelial cells (HUVECs) and human mesenchymal stem cells (MSCs) (Takebe et al., 2013) (Fig. 1D), or from HE, endothelial cells and MSCs generated entirely from human iPSCs, increasing the translational appeal of this particular organoid model (Takebe et al., 2017). Co-culture significantly increased the expression of key hepatic genes compared with hepatocytes that were differentiated alone. To test the functionality of the hPSC-liver buds, transplantation studies were performed in immunocompromised mice (Takebe et al., 2013). Notably, compared with adult hepatocytes and hPSC-derived hepatocytes that were differentiated in isolation, the hPSC-liver buds produced higher sustained levels of albumin, suggesting functional maturation of the hepatocytes within the liver bud. Moreover, hPSC-liver buds outperformed transplanted adult hepatocytes when the mice were challenged in a liver failure model.

The flexibility of liver bud organoids was leveraged to interrogate the contribution of paracrine signals in directing liver bud organoid formation and maturation (Asai et al., 2017). This was achieved using a transwell culture system to physically separate the cell lineages involved in co-culture, while permitting crosstalk via secreted molecules through a semipermeable membrane (Fig. 1D). Paracrine signals from either HUVECs or MSCs were capable of inducing hPSC-HE to produce more albumin and α1-antitrypsin, which indicates the increased functionality of resulting hepatocyte-like cells compared with non-co-cultured controls. When HUVECs and MSCs were grown together, this paracrine effect was dampened. Proteomic analysis was used to identify angiotensinogen, α-2 macroglobulin and plasminogen as secreted molecules that were present in the co-culture media when hepatic endoderm was co-cultured with either HUVECs or MSCs and therefore may play a role in hepatocyte maturation. This work presented an *in vitro* platform to systematically investigate paracrine signaling and identify key maturation factors of hepatic endoderm into hepatocytes.

Further interrogation of hPSC-liver buds at the single cell level has provided additional high-resolution insights into the directed differentiation of 3D liver buds (Camp et al., 2017). Single cell RNA sequencing followed by pseudotime ordering of cells across the hepatocyte differentiation process revealed the emergence of a maturation signature in differentiating hepatocytes as they underwent 3D organization in the co-culture conditions. These signatures were distinct from the 2D monotypic culture, despite both culture methods yielding albumin-expressing hepatocyte-like cells. A comparison of cells resulting from each method with those from primary liver tissue further distinguished cells resulting from 2D or 3D co-culture, with 3D-derived cells possessing a transcriptional signature most similar to human fetal, rather than adult, hepatocytes. Collectively, these studies clearly demonstrated that 3D co-culture with endothelial and mesenchymal cells improved existing models of hPSC-derived hepatocyte-like cells.

## Approaches for promoting organoid maturation

**Fig. 2.**
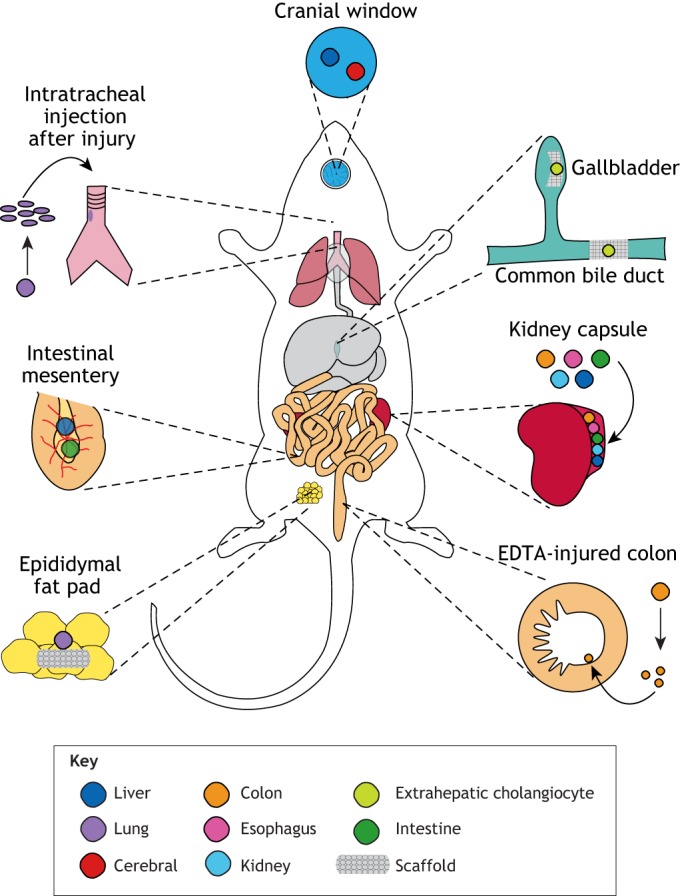
**Orthotopic and ectopic transplantation of human organoids.** Both hPSC and primary tissue has been transplanted into immunocompromised mice. The colors represent different types of organoids, both primary and/or hPSC-derived, that have been transplanted into the indicated locations. Orthotopic transplantation refers to transplantation into the analogous *in vivo* location, whereas ectopic transplantation refers to transplantation into a region outside of the native *in vivo* environment (i.e. beneath kidney capsules, within fat pads).

Organoids exhibit restricted growth *in vitro* as they must rely on diffusion of oxygen and exogenous nutrients to survive. In addition, many hPSC-derived organoid models more closely resemble an immature tissue and fail to mature regardless of duration in culture. However, transplantation of organoids into various sites in mammalian hosts have resulted in organoid maturation and increased tissue functionality (Fig. 2). Organoids have been transplanted both orthotopically (i.e. into the analogous *in vivo* location) and ectopically (i.e. in a region outside of the native *in vivo* environment) and both transplantation methods are permissive for organoid maturation (Dye et al., 2016; Finkbeiner et al., 2015b; Sugimoto et al., 2018; Takebe et al., 2013; van den Berg et al., 2018). These studies have demonstrated that organoid models are capable of maturing when provided with additional cues from the *in vivo* environment, thereby increasing their appeal to study human organogenesis and for use as pre-clinical models or in regenerative medicine. Here, we discuss methods to improve organoid complexity and maturation through transplantation *in vivo*.

### Ectopic transplantation of organoids

Many experiments have transplanted organoids into immunocompromised mice for maturation. Typically, organoids were transplanted into highly vascularized sites that are amenable for organoid engraftment and growth for up to several months, without obstructing necessary murine organ function. Such sites include epididymal fat pads (Dye et al., 2016) and underneath kidney capsules (Finkbeiner et al., 2015a,b; Múnera et al., 2017; Trisno et al., 2018; Tsai et al., 2017; Watson et al., 2014; Workman et al., 2016). Indeed, the complexity of co-cultures described earlier can be further enhanced by transplantation (Schlieve et al., 2017; Takebe et al., 2013; Workman et al., 2016).

HIOs are an example of an organoid model that has shown significant maturation after kidney capsule transplantation for several weeks (Finkbeiner et al., 2015b; Watson et al., 2014). Both epithelium and mesenchyme of transplanted HIOs (tHIOs) displayed enhanced organization, with the emergence of a properly patterned villus-crypt architecture within the epithelium that was not apparent before transplantation. Further, smooth muscle actin (SMA)- and platelet derived growth factor receptor alpha (PDGFRA)-expressing mesenchymal lineages became organized in a manner that was indistinguishable from the native human intestine (Finkbeiner et al., 2015b). Vascularization by mouse blood vessels supported significant growth of transplanted HIOs up to 100-fold in size (Watson et al., 2014). Recently, HIOs were successfully transplanted into the intestinal mesentery and demonstrated similar growth and maturation hallmarks as those transplanted beneath the kidney capsule (Cortez et al., 2018; Finkbeiner et al., 2015a). The intestinal mesentery is therefore a complementary transplantation site that may be more physiologically relevant for future translational studies owing to its close proximity to the native gut.

hPSC-derived lung organoids (HLOs) are another *in vitro* model that has shown improved tissue architecture and maturation upon transplantation. Interestingly, unlike several other organoid models, HLOs did not survive transplantation beneath the kidney capsule (Dye et al., 2016). Rather, a microporous polylactide-co-glycolide (PLG) scaffold, which is commonly used to transplant pancreatic beta cells (Gibly et al., 2011; Hlavaty et al., 2014), was used to support HLO transplantation. The PLG scaffold served as a physical niche to support survival and maturation of transplanted HLOs, which displayed enhanced epithelial organization, morphology and differentiation into proximal airway (i.e. trachea/bronchi) lineages that more closely resembled the mature human proximal airways than HLOs grown *in vitro*. This result suggested that *in vivo* cues are required, along with the scaffold, to support HLO maturation. Although alveolar lineages were present in the *in vitro* gown HLOs, alveolar cell types and structures were not supported in transplanted HLOs (Dye et al., 2015, 2016). Notably, HLOs must be transplanted within the epididymal fat pad, because the kidney capsule compartment was not large enough to accommodate the scaffold. Despite the difference in transplantation location, similar to intestinal organoids, HLOs were extensively vascularized by host vessels and both the epithelial and mesenchymal components matured after *in vivo* growth (Dye et al., 2016; Finkbeiner et al., 2015b; Watson et al., 2014). This study demonstrated the requirement of a physical niche to support survival, differentiation and maturation of transplanted HLOs.

These studies demonstrated that vascularization is key for engraftment, growth and survival of transplanted organoid tissues, but vascularization may be slow and/or inefficient for organoid systems that do not possess vasculature before transplantation. Liver bud organoids with pre-existing endothelial cells (HUVECs) engrafted and established blood flow within only a few days (Takebe et al., 2013). In addition, transplantation into a cranial window allowed for live imaging of organoids and demonstrated that HUVECs formed a stable vascular connection with the host.

Data suggests that most organoid models do not inherently contain vasculature; however, endogenous vascular cells are present within hPSC-derived kidney organoids. This likely resulted from the directed differentiation approach, which pushes hPSCs into an intermediate mesoderm fate, generating nephrogenic lineages as well as endothelial cells (Freedman et al., 2015; Takasato et al., 2015, 2016). After transplantation beneath the kidney capsule, kidney organoids became extensively vascularized by both host and organoid endothelial cells that persisted for at least 2 weeks of transplantation (van den Berg et al., 2018). Moreover, use of an abdominal imaging window allowed for live imaging of the organoids throughout transplantation. Infusion of fluorescent dextran in the blood allowed the visualization of blood flow and helped to demonstrate that glomerular structures within the kidney organoid, which function to filter urine from the blood, became vascularized. This led to improved glomerular maturation, including deposition of glomerular basement membrane and a fenestrated endothelium within the transplanted organoids.

### Orthotopic transplantation of organoids

Although ectopic transplantation is a valuable tool to allow for organoid maturation, there is interest in orthotopically transplanting organoids or organoid-derived cell types to advance our understanding of *in vivo* niches in regulating organoid maturation, as well as increasing the translational potential of organoid models for organ replacement therapies. Injury models have been used across various organ systems to create niches that are permissive for both primary and hPSC-derived organoid engraftment in physiologically relevant sites in adult mouse models (Huch et al., 2015; Miller et al., 2018; Sampaziotis et al., 2017; Sugimoto et al., 2018). For example, recent efforts have successfully orthotopically transplanted human primary tissue-derived colonic epithelial organoids into the murine colon after disruption of the endogenous epithelium (Sugimoto et al., 2018). Here, it was demonstrated that a significant area of injury was required to create a niche large enough to promote engraftment and persistence of human tissue over several months. Smaller injuries also allowed for engraftment, but ultimately engrafted human tissue was lost over time. Lineage tracing of the intestinal stem cells using a genome engineered intestinal stem cell-specific LGR5-CreER and a lineage reporter in human colonic organoids revealed important species-specific differences in cycling time between mouse and human colonic stem cells (Sugimoto et al., 2018). This insight into the intestinal stem cell niche would not have been possible with ectopic transplantation.

Similar injury-engraftment approaches have been used to introduce hPSC-derived human lung bud tip epithelial progenitor organoids into an injured adult mouse lung (Miller et al., 2018; Miller et al., 2019). Human lung bud tip progenitors can engraft into the trachea and proximal airways of the mouse lung following chemically induced bronchiolar injury. Moreover, these cells demonstrated multi-lineage differentiation potential into several proximal lineage phenotypes including mucus-producing cells, ciliated cells and neuroendocrine cells after 6 weeks post injury. Notably, these progenitors showed *in vitro* alveolar differentiation capabilities, but such lineages were not observed, likely because cells did not engraft in alveolar areas in which there was no injury in this particular injury model. This result highlights the importance of the *in vivo* microenvironment in mediating organoid progenitor differentiation (Miller et al., 2018). Finally, extrahepatic cholangiocyte primary tissue-derived organoids seeded on scaffolds were able to engraft into the epithelium of the gallbladder and common bile ducts to functionally rescue mouse models of extrahepatic biliary injury (Sampaziotis et al., 2017).

Human hPSC-derived cerebral organoids have also been successfully transplanted into the cerebrum of adult mice with robust organoid engraftment and host survival rates, despite the invasiveness of the procedure (Mansour et al., 2018). In comparison with age-matched *in vitro*-grown cerebral organoids, transplanted cerebral organoids contained a higher proportion of mature neuronal cell fates that included astrocytes and oligodendrocytes, compared with relatively immature neuronal precursors observed *in vitro*. In addition, axon projections were shown to invade various regions of the host brain, and marker analysis suggested that these human organoid-derived axons were forming synaptic connections with the murine neurons. Initial studies demonstrated that transplanted cerebral organoids responded to external calcium stimulation in a synchronized pattern most similar to a developing, rather than mature, brain. Therefore, although this was an impressive advancement in cerebral organoids, the current transplantation approaches result in only partial maturation (Mansour et al., 2018). Efforts have also derived brain organoids with endothelial cells from hPSCs that could be co-cultured *in vitro* for up to 5 weeks and transplanted into a murine host for at least 2 weeks, demonstrating proof-of-principle for generating human vasculature in transplanted brain organoids (Pham et al., 2018).

Collectively, the establishment of orthotopic transplantation approaches enables the study of organoid maturation within the native tissue microenvironment, and at the same time brings researchers a step closer towards translational applications for organoid technologies. In both ectopic and orthotopic approaches, it has been challenging to identify the exact factors in the *in vivo* host environment that are responsible for organoid maturation. Moving forward it will be important to begin tackling these *in vivo* interactions in order to apply principles of maturation to create mature organoids in the dish. To address these questions, future experiments could generate organoids from a CRISPR knockout library or screen a small molecule library to independently assess factors in maturation.

### Engineering-transplantation approaches to increase organoid maturity

Intestinal organoids are a promising source of replacement tissue for conditions such as short bowel syndrome, but clinical implementation will require increases in size and maturity to produce functional intestinal tissue. One approach to address this issue employed HIOs that were seeded on scaffolds to produce tissue-engineered small intestine (TESI) (Finkbeiner et al., 2015a). HIOs were seeded on acellular porcine intestinal matrices as well as synthetic scaffolds comprising polyglycolic/poly L lactic acid (PGA/PLLA). Using this approach, HIOs successfully reseeded the acellular matrices *in vitro* but did not persist or retain intestinal identity when transplanted into immunocompromised mice. The poor performance of acellular matrices could be due to removal of instructive cues during decellularization or blockage of vasculature infiltration to HIOs by the matrix. However, HIOs that were seeded on the synthetic matrices thrived *in vivo* and exhibited epithelial maturation, resulting in tissue that resembled the adult intestine. These tubular-shaped scaffolds are a promising approach to seed multiple HIOs and generate longer, more mature intestinal tissue *in vitro*. Although the TESI described here lacked key intestinal cell types such as an enteric nervous system, this can be overcome as described above (Schlieve et al., 2017; Workman et al., 2016).

A more recent approach to generate scalable, mature intestinal tissue from HIOs incorporated mechanical strain using lengthening springs (Poling et al., 2018). Mechanical strain has been shown to play a role in intestinal development (Kurpios et al., 2008; Savin et al., 2011; Shyer et al., 2015, 2013), and spring or stretch-based lengthening devices have been designed as treatment methods for short bowel syndrome (Demehri et al., 2015, 2014; Ralls et al., 2012; Rouch et al., 2016; Stark et al., 2012; Sueyoshi et al., 2013). In this approach, HIOs were transplanted into immunocompromised mice for 10 weeks. After this time, compressed nitinol springs were surgically inserted into the transplanted HIOs for an additional 14 days. The transplanted HIOs that had undergone strain exhibited increased intestinal length, larger villi and deeper crypts and appeared to be more mature based on global gene signatures, compared with transplanted HIOs without springs. These studies highlight bioengineering approaches to promote clinical applications of HIOs and demonstrate the importance of both biological and mechanical cues in promoting organoid maturation.

## Bioengineering 3D environments to increase reproducibility and clinical use of organoid technology

One of the major challenges facing organoid research is reproducibility and limited control over the 3D self-organization process (Huch et al., 2017; Spence, 2018). Currently, the same experimental conditions may yield organoids with variations in cellular composition, architecture, size and shape that limit modeling of development and clinical translation (Huch et al., 2017). A major cause of variation in organoid cultures is animal-derived ECM products such as Matrigel, which are standard for culturing many organoid models including intestine, lung and liver (Dye et al., 2015; Miller et al., 2018; Spence et al., 2010; Takebe et al., 2013). Natural ECMs limit reproducibility and control over organoid formation as they are prone to batch-to-batch variability, comprised of a poorly defined protein and growth factor composition, and cannot be easily modified to control biophysical and biochemical matrix properties. In addition, animal-derived ECM limits clinical translation as it is a xenogeneic material and poses a risk for pathogen transfer (Czerwinski and Spence, 2017).

To address these limitations, bioengineers are working to create well-defined systems for organoid culture. Given that both biochemical and mechanical/physical cues drive organogenesis (Kurpios et al., 2008; Savin et al., 2011; Shyer et al., 2015; Walton et al., 2012), it is important that matrix cues such as mechanical stiffness, degradability and adhesive ligand presentation can be independently modulated. This will maximize the ability to model the complex interplay between the physical environment, cell behavior and organ formation using organoid models. In addition, it is likely that each organ will require a unique biochemical and physical niche to support optimal development. Here, we discuss bioengineering strategies that have enhanced reproducibility, experimental control and/or precision, and clinical application of organoids. Organ-on-a-chip advances are not discussed here, and have been reviewed elsewhere (Ronaldson-Bouchard and Vunjak-Novakovic, 2018; Zhang et al., 2018a). Future work may focus on combining organ-on-a-chip technologies with organoids to model crosstalk between organ systems.

### Defined ECM-mimetic hydrogels

The importance of choosing an appropriate ECM or ECM-mimetic to control organoid properties was demonstrated in a study that compared tissue-derived intestinal epithelial organoids grown in floating collagen I gels with Matrigel-grown organoids (Sachs et al., 2017). In collagen, but not Matrigel, organoids aligned, fused and formed macroscopic hollow tubes with a single enclosed lumen and budding crypt-like domains, more closely resembling the native architecture of the intestine. Collagen gel contraction by organoids may enable tube formation. This study demonstrates that simply changing ECM constituents can increase control over organoid formation and/or organization and presents a system to model the intestine on a larger scale. These experiments were performed using mouse organoids, but the importance of the ECM environment on organoid behavior is applicable to human systems as well. In addition, defined collagen I matrices have been used to culture intestinal epithelial cells supplemented with Wnt3a to promote a repairing epithelial phenotype, further demonstrating the applicability of defined hydrogel systems (Yui et al., 2018).

Another recent study used defined fibrin hydrogels that were supplemented with adhesive cues to increase experimental control over epithelial organoid culture (Broguiere et al., 2018). Experiments on mouse intestinal epithelial organoids in fibrin revealed that organoid formation depended on matrix properties, as increased stiffness or decreased access to the adhesive motif RGD reduced organoid formation. Supplementation with laminin, the major component of Matrigel, led to organoid yields in fibrin that were comparable with those in Matrigel. The fibrin/laminin hydrogel system was successfully applied to culture human epithelial organoids derived from small intestine, liver and pancreas.

**Fig. 3.**
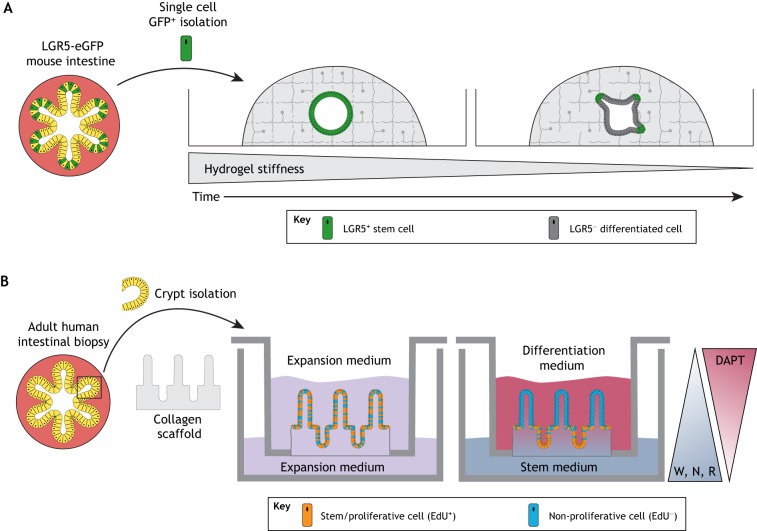
**Bioengineered 3D environments support human intestinal organoid cultures.** (A) Mechanically dynamic PEG hydrogels support intestinal epithelial organoid formation. LGR5+ intestinal stem cells were expanded in PEG hydrogels with an initial stiffness that softened over time via hydrolytic degradation to accommodate differentiation. (B) Generation of polarized crypt/villus structures through a combination of micropatterning and signaling gradients. Isolated human primary intestinal crypts were cultured on a microengineered collagen scaffold to produce crypt/villus architecture that became patterned into proliferative/differentiated domains through exposure to differential signaling gradients across a transwell system. DAPT, N-[N-(3,5-difluorophenacetyl)-L-alanyl]-S-phenylglycine t-butyl ester (γ-secretase inhibitor); W, Wnt; N, noggin; R, R-spondin.

In addition to collagen and fibrin/laminin, a recent landmark study demonstrated that chemically defined polyethylene glycol (PEG) hydrogels could be used as an ECM replacement to culture intestinal epithelial organoids (Gjorevski et al., 2016). These hydrogels served as a synthetic ECM-mimetic to exert better control over growing organoids and to reduce reliance on animal-derived matrices. By working with PEG, a biologically inert hydrogel that possesses no inherent ability for cells to adhere to or degrade the matrix unless otherwise modified, variables including matrix stiffness, degradation properties and adhesiveness were independently modulated. In order to determine the effects of these properties on intestinal stem cell proliferation, organoid formation and cellular differentiation, PEG hydrogels were modified with proteolytically degradable crosslinkers and cell-adhesive peptides. This work identified that, although a stiff matrix was optimal for initial intestinal stem cell expansion, maintaining cells in a stiff matrix prohibited differentiation, suggesting that epithelial differentiation required a softer matrix. Based on these observations, a mechanically dynamic matrix was created to support stem cell expansion and then adapt to permit organoid differentiation. These dynamic hydrogels exhibited an initial stiffness that was optimized for stem cell expansion but were hydrolytically active and therefore able to soften over time to permit cellular differentiation by alleviating compressive forces (Fig. 3A). Although these hydrogels were optimized using mouse intestinal epithelial organoids, the resulting matrices were successfully used to culture human intestinal epithelial organoids as well.

Recent work has also demonstrated that defined PEG hydrogels can be used to replace Matrigel for the culture of hPSC-derived human intestine and lung organoids (Cruz-Acuña et al., 2017). PEG hydrogels were modulated to determine specific mechanical properties and adhesive ligand presentation that optimized HIO formation, thus identifying a stiffness range and adhesive motif (RGD) that supported HIO viability. HLOs were viable in PEG hydrogels that were optimized for HIO culture, but matrix properties such as stiffness and adhesive cues may need to be optimized for different organoid systems. In addition, PEG hydrogels served as an injection vehicle to deliver HIOs to injured mucosa, providing an *in situ* polymerized gel that improved HIO engraftment, compared with HIOs that were injected without a delivery vehicle. Interestingly, in the case of hPSC-derived HIOs, which comprise both an inner epithelium and an outer mesenchymal layer, adhesive cues and matrix degradability are dispensable, as HIOs have been cultured in alginate, a natural biologically inert polymer which lacks cell-instructive signals (Capeling et al., 2019).

Chemically defined PEG hydrogels have also been used to control organization of mouse pluripotent stem cells into neuroepithelial cysts, or organoids that mimic neural tube morphogenesis (Ranga et al., 2016). A high-throughput system was used to screen factors (mechanical properties, adhesive ligands and degradability) that are necessary for neuroepithelial morphogenesis in PEG hydrogels. This study used mouse organoids, but the methodology may be more broadly applicable to human systems as well. Notably, the ideal properties identified for neural tube morphogenesis did not match the properties tuned for intestinal development (Gjorevski et al., 2016; Ranga et al., 2016), although differences may arise from comparison between human and mouse systems. Nonetheless, this suggests that using Matrigel as a ‘one-size-fits-all’ matrix for all organoid systems is suboptimal. Interestingly, neuroepithelial colonies in Matrigel exhibited more heterogeneous colony sizes and morphology compared with colonies in PEG hydrogels, whereas a greater proportion of cysts in PEG became properly polarized compared with those in Matrigel. This highlights the utility of defined matrices to increase reproducibility in organoid cultures.

### Engineered control over local matrix properties

Although these defined matrices offer improved control over organoid formation, current hydrogel systems have largely focused on control over bulk properties and have not provided spatiotemporal control over local mechanical cues or signaling gradients. Recently, micropatterned hydrogels were used to provide controlled positional information to intestinal epithelial cells and guide organization of crypt/villus structures that mimic the structure of the native intestine (Wang et al., 2017) (Fig. 3B). In this system, human small intestine crypt fragments were cultured on collagen gels that were micromolded into crypt/villus domains, creating a pattern for cell growth with distinct luminal and basal fluid reservoirs that enabled spatial control over the local signaling environment by application of different media conditions on either side of the reservoir. This strategy produced intestinal epithelium arranged in proliferative crypt zones with differentiated villus domains based on the application of signaling gradients. A similar strategy has also been used to micropattern crypts derived from human colonic epithelium (Wang et al., 2018). Although micropatterned techniques enable control over the spatial organization and signaling environment of 3D cell culture systems, they do not promote the self-organization that is provided within organoid model systems to properly model development, especially as this system lacked a mesenchymal component. Future work may focus on the development of dynamic hydrogels to spatiotemporally control matrix mechanical and biochemical properties in order to better control organoid development.

Interestingly, a recent study combined these two strategies of organoid self-assembly with bioengineered constructs to guide the formation of human brain organoids (Lancaster et al., 2017). Fiber microfilaments comprising poly(lactide-co-glycolide) (PLGA) were used as floating scaffolds to pattern embryoid bodies from hPSCs. This method enabled the shaping of brain organoids from the inside at an early stage. By increasing the surface-area-to-volume ratio with these microfilament scaffolds, reproducible induction of neuroectoderm from micropatterned embryoid bodies was observed. Patterned embryoid bodies were then transferred to Matrigel droplets for expansion into engineered cerebral organoids (enCORs). This work demonstrates the potential for bioengineered micropatterned constructs to guide and control organoid self-assembly.

Although bioengineering-defined environments for organoid culture present a promising approach to better control organoid development and increase reproducibility, there may still be uncontrollable elements in the differentiation process that are unrelated to the growth matrix. For example, recent work has revealed heterogeneity in hindgut spheroids, the precursor to HIOs, that contributes to inefficient organoid formation and may additionally contribute to variation in organoid phenotype (Arora et al., 2017). Process engineering was used to develop a pipeline that sorted spheroids based on size and identified certain phenotypes predisposed for growth into HIOs in order to increase organoid yield from this selected population. This study demonstrated the necessity of controlling all aspects of organoid formation, and highlights a methodology to engineer early aspects of HIO maturation.

## Conclusion

Research efforts have begun to shift away from simply generating new organoid model systems toward improving fidelity, reproducibility and developing new applications that build from existing systems to explore unanswered questions in human development and disease. Co-cultures have allowed researchers to successfully integrate missing cell lineages including endothelial cells, immune cells and neurons into a few *in vitro* systems, thereby increasing complexity. However, it is important to highlight that organization of exogenously added cell types often does not resemble or function as the mature *in vivo* tissue. Instead, transplantation into murine hosts is often needed to enhance organization within the organoid. Functional maturation of organoids that result from *in vivo* engraftment into an immunocompromised murine host has also opened the door for investigating new areas; for example, future work may focus on understanding the mechanisms by which in the *in vivo* environment promotes maturation so that organoids can be coaxed to mature without the need for animal hosts. Moreover, improved control over the *in vitro* signaling environment and physical and/or mechanical environment using tunable matrices will allow for improved spatiotemporal control over differentiation to better mimic developmental processes.

Insights gleaned from single cell genomic characterization can also be leveraged to improve organoid models. For example, work in mouse intestinal organoids directly compared Paneth cells that had been derived from primary intestinal epithelial organoids with those found in the *in vivo* tissue (Mead et al., 2018). Comparison of *in vitro*-derived and *in vivo* Paneth cells revealed differential expression of antimicrobial peptides and defensin proteins. Previous studies in animal models suggest that modulating Notch and Wnt signaling could improve differentiation of intestinal stem cells into the Paneth cell fate. Therefore, the authors hypothesized that increasing expression of antimicrobial peptides in these organoids in order to more closely resemble *in vivo* Paneth cells could be achieved by activating Wnt and inhibiting Notch signaling using small molecules in the growth media. These new culture conditions generated intestinal organoids containing Paneth cells that more faithfully represented *in vivo* Paneth cells based on transcriptional and functional characterization. This work nicely demonstrates how single-cell profiling can be used to implement targeted approaches that improve organoid fidelity.

Moving forward, continued efforts to compare cells in human organs with cells in organoids both functionally and at the molecular level, combined with approaches to improve organoid complexity and to exert fine control over the system using engineering solutions, will allow the community to build more realistic organoid tools. These improved tools will undoubtedly further our fundamental understanding of human biology in the contexts of development, homeostasis and disease.
